# Metabolic Glycoengineering Enables the Ultrastructural Visualization of Sialic Acids in the Glycocalyx of the Alveolar Epithelial Cell Line hAELVi

**DOI:** 10.3389/fbioe.2020.614357

**Published:** 2021-01-14

**Authors:** Raphael Brandt, Sara Timm, Jacob L. Gorenflos López, Jubilant Kwame Abledu, Wolfgang M. Kuebler, Christian P. R. Hackenberger, Matthias Ochs, Elena Lopez-Rodriguez

**Affiliations:** ^1^Institute of Functional Anatomy, Charité – Universitätsmedizin Berlin, Berlin, Germany; ^2^Core Facility Electron Microscopy, Charité – Universitätsmedizin Berlin, Berlin, Germany; ^3^Department Chemical Biology, Leibniz-Forschungsinstitut für Molekulare Pharmakologie (FMP), Berlin, Germany; ^4^Department of Chemistry, Humboldt Universität zu Berlin, Berlin, Germany; ^5^Institute of Physiology, Charité-Universitätsmedizin Berlin, Berlin, Germany; ^6^German Center for Lung Research (DZL), Berlin, Germany

**Keywords:** glycocalyx, sialic acid, electron microscopy, metabolic glycoengineering, bioorthogonal labeling, alveolar epithelium, hAELVi, huAELVi

## Abstract

The glycocalyx—a plethora of sugars forming a dense layer that covers the cell membrane—is commonly found on the epithelial surface of lumen forming tissue. New glycocalyx specific properties have been defined for various organs in the last decade. However, in the lung alveolar epithelium, its structure and functions remain almost completely unexplored. This is partly due to the lack of physiologically relevant, cost effective *in vitro* models. As the glycocalyx is an essential but neglected part of the alveolar epithelial barrier, understanding its properties holds the promise to enhance the pulmonary administration of drugs and delivery of nanoparticles. Here, using air-liquid-interface (ALI) cell culture, we focus on combining metabolic glycoengineering with glycan specific electron and confocal microscopy to visualize the glycocalyx of a recently immortalized human alveolar epithelial cell line (hAELVi). For this purpose, we applied different bioorthogonal labeling approaches to visualize sialic acid—an amino sugar that provides negative charge to the lung epithelial glycocalyx—using both fluorescence and gold-nanoparticle labeling. Further, we compared mild chemical fixing/freeze substitution and standard cytochemical electron microscopy embedding protocols for their capacity of contrasting the glycocalyx. In our study, we established hAELVi cells as a convenient model for investigating human alveolar epithelial glycocalyx. Transmission electron microscopy revealed hAELVi cells to develop ultrastructural features reminiscent of alveolar epithelial type II cells (ATII). Further, we visualized extracellular uni- and multilamellar membranous structures in direct proximity to the glycocalyx at ultrastructural level, indicating putative interactions. The lamellar membranes were able to form structures of higher organization, and we report sialic acid to be present within those. In conclusion, combining metabolite specific glycoengineering with ultrastructural localization presents an innovative method with high potential to depict the molecular distribution of individual components of the alveolar epithelial glycocalyx and its interaction partners.

## Introduction

### The Alveolar Lining Layer as Integral Part of the Blood-Air-Barrier

The lung alveolar epithelium constitutes the first cell lining that atmospheric oxygen has to pass for oxygen uptake in the lungs. Thus, its morphology needs to meet criteria for optimal gas exchange. While the majority of the alveolar epithelial surface is comprised of extended alveolar epithelial type I cells (ATI) whose flattened architecture allows for efficient gas exchange, the majority of alveolar epithelial cells belongs to the smaller alveolar epithelial type II cells (ATII) (Hsia et al., 2016). The multifunctional ATII are cuboidal in shape, have important secretory capacity and are further considered as proliferative precursors capable of differentiation into ATI, most notably upon lung injury (Matthay et al., 2019).

During exhalation, physically relevant pressure differences put the smaller alveoli at constant risk of collapse. Keeping the alveoli homogeneously open in all states of respiration is a function that has been attributed to a continuous liquid lining layer covering the alveolar epithelium. The alveolar lining layer comprises two phases: a liquid hypophase and an upper film composed of a lipid-protein mixture called lung surfactant (surface active agent) (Pattle, 1955). Surfactant is best known for its ability to cause a surface-dependent decrease of surface tension at the air-liquid interface, which prevents collapse of the alveolar space, especially during exhalation. Extending its biophysical mode of action, additional functions have been attributed to surfactant, including a role in immunomodulation (Wright, 2005; Han and Mallampalli, 2015). Surfactant has been in the center of fruitful translational research for the last decades (Owen et al., 2017), resulting in beneficial pharmaceutical treatments for preterm children born at a stadium of immature lung development.

### The Alveolar Glycocalyx—Little Is Known

The underlying liquid hypophase acts as a space for assembly and recycling of surfactant precursor and degradation products, respectively, but also consists of an apical sugar-rich cell surface coat called the glycocalyx. The latter has recently been proposed to interact with surfactant components (Ochs et al., 2020). The epithelial surface of lumen forming organs is characteristically rich in glycocalyx, providing overlapping but also highly tissue specific functions. The chemical complexity of the glycocalyx is immense, as are the different glycans forming it (Mockl, 2020). Not only the order at which different monosaccharides can be combined to form poly-antennary polymers of different lengths is of relevance, but also the types of glycosidic bonds covalently linking the chain of monosaccharides. Two monosaccharides can be linked *via* glycosidic bonds between the anomeric hydroxyl of the first unit, and either one of several possible hydroxyl groups of the second, which vastly increases the combinatory potential (Laine, 1994). In addition, chemical modifications such as oxidation of hydroxyl groups, sulfation, N-acetylation, sialylation, to name a few, extend the spectrum of potential functional properties of glycans, for instance by adding specific charges. Given this steric and chemical complexity and its position at the cell periphery, not only the structural organization but also the regulatory potential of the glycocalyx is increasingly recognized in the field (Varki, 2017). Sialic acids serve as receptors for the attachment of many viruses to host cells (Matrosovich et al., 2015) and have been shown to determine pulmonary endothelial barrier integrity (Cioffi et al., 2012). Also, exposure to cigarette smoke was shown to influence levels of sialylation in an alveolar epithelial cancer cell model (Wielgat et al., 2018).

Despite upcoming interest in the glycocalyx of the alveolar epithelium, its structure and functions remain largely unexplored (reviewed in Ochs et al., 2020). This is first, attributable to the lack of physiologically relevant and cost effective *in vitro* models. Secondly, traditional methods to study biological systems are of limited use when studying the glycocalyx (reviewed by Mockl, 2020). Thirdly, the focus of research of the alveolar lining layer had strictly been on surfactant over the last decades, thereby neglecting the glycocalyx.

### Using Metabolic Glycoengineering to Label Sialic Acids

**FIGURE 1:**
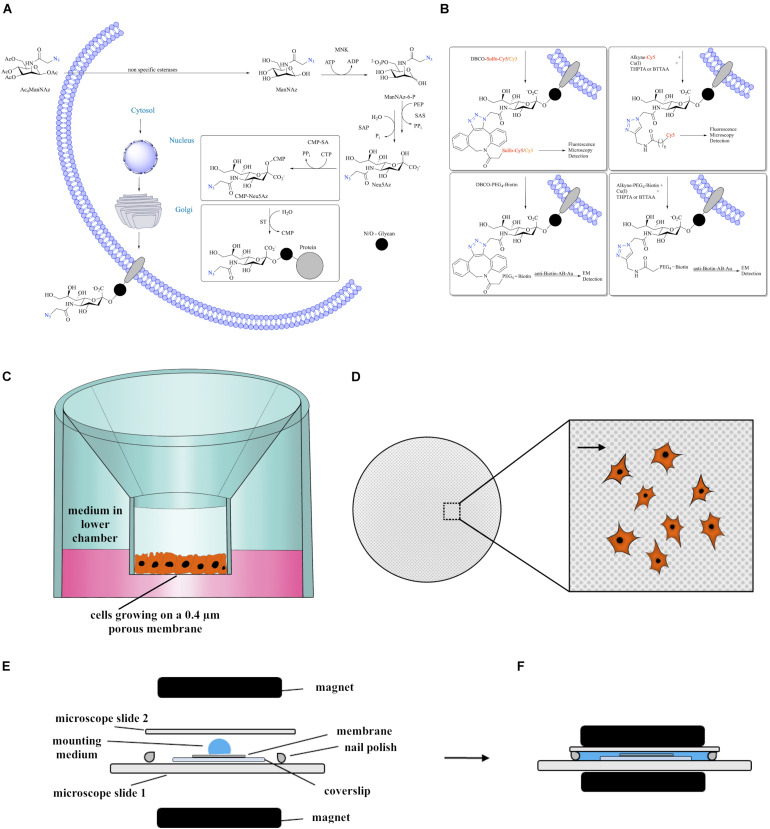
**(A)** Introduction of Ac_4_ManNAz to the sialic acid biosynthesis pathway. **(B)** Different approaches of labeling azide-modified sialic acid that were successfully applied in this study and the and the method of detection. These encompass biorthogonal reactions via CuAAC or copper-free SPAAC linked to fluorophores or biotin, enabling either fluorescence microscopy or, upon immunogold labeling using antibodies against biotin, electron microscopy. **(C)** Scheme showing cells growing at ALI using a transwell membrane inset that is placed in a well of a 12-well plate filled with medium. **(D)** Scheme of the membrane as seen from the top. Large Inset displays higher magnification of the porous membrane with plated cells. Arrowhead points to a 0.4 μm pore. **(E,F)** Schematic visualization of the embedding procedure used for fluorescence microscopy. The magnets are applied carefully in order to flatten the membrane.

Metabolic glycoengineering (MGE) is a method used to modify glycan structures by treating cells with unnatural derivatives of monosaccharides. Especially, sialic acid modification using *N*-acetyl-mannosamine (ManNAc) derivatives have become a standard methodology (Wratil et al., 2016). Sialic acids are nonose monosaccharides primarily found as terminal components of glycans. They play crucial roles in cellular processes such as extrinsic and intrinsic cell-cell communication as well as in defense (Schauer and Kamerling, 2018). MGE is based on the promiscuity in the sialic acid biosynthesis pathway and has enabled the introduction of a variety of bioorthogonal reporters into the sialic acid component. For the incorporation of azides, acetylated *N*-azidoacetyl-mannosamine (Ac_4_ManNAz) developed by the Bertozzi group is the most prominent example (Jacobs et al., 2000; Baskin et al., 2007; Moller et al., 2011). After entering the cell, non-specific esterases cleave the acetyl groups and ManNAz is processed by the promiscuous enzymes of the sialic acid biosynthesis pathway (Figure 1A) to CMP-Neu-5-Az. The latter is used by sialic acid transferases in the Golgi to modify the glycans of glycoproteins by adding Neu-5-Az (Wratil et al., 2016). The glycosylated proteins become, for the most part, tethered to the cell surface.

The introduction of azides to the cell surface *via* MGE enables the employment of bioorthogonal reactions for the specific labeling *in vitro* and *in vivo*, which do otherwise not interact with the biochemistry of the cells (Baskin et al., 2007; Kenry and Liu, 2019). Here, we employed this method by bioorthogonally reacting azide-containing sialic acids obtained from ManNAz with alkynes linked to fluorophores or biotin. The latter was subsequently detected by anti-biotin-antibodies coupled to gold nanoparticles (anti-Biotin-AB-Au). We applied two bioorthogonal labeling approaches: Cu(I) catalyzed azide-alkyne cycloaddition (CuAAC) with THPTA or BTTAA (see Abbreviations list for chemical names) as stabilizing additives or strain-promoted-azide-alkyne cycloaddition (SPAAC), which is copper-free and uses cyclooctynes such as dibenzocyclooctyne (DBCO) (Figure 1B).

### Visualizing the Glycocalyx—Electron Microscopy as a Method of Choice

The alveolar lining layer has an average height of 200 nm in rat lungs (Bastacky et al., 1995). The size of the glycocalyx, which constitutes the bottom part of the lining layer, is therefore regularly below the diffraction limit of light (∼250 nm). Thus, conventional optical microscopy is limited in accurately assessing glycocalyx dimensions, and resolving fine structures below 25 nm can exclusively be performed by electron microscopy (EM) methods. EM requires elaborate sample preparation, and there is ongoing debate if and how the required treatments affect the ultrastructural preservation of the glycocalyx (Ebong et al., 2011; Hegermann et al., 2016; Chevalier et al., 2017). Furthermore, the molecular identity of glycans is not depicted by conventional EM, despite dynamic progress in this regard (Broecker et al., 2015).

Here, we overcame some of these limitations by combining MGE with EM sample preparation based on mild chemical fixation followed by freeze substitution (FS) and postembedding immunogold labeling to determine sialic acid localization within the glycocalyx of alveolar epithelial cells at ultrastructural resolution. This is not only a methodically achievement in itself but also guides interpretation of MGE-based light microscopy imaging. We compared the ultrastructural preservation of the glycocalyx upon FS to conventional and glycan labeling EM protocols, the latter using Alcian blue. In addition, our work adds new insights into specific morphological features of the recently developed hAELVi cell line (Kuehn et al., 2016), e.g. microvilli dense regions and extracellular membranous structures, both of which show a marked sialic acid distribution. In summary, we established hAELVi cells as a model for the study of the alveolar epithelial glycocalyx.

## Materials and Equipment

A list of reagents and equipment required for cell culture, confocal and electron microscopy preparation is provided in Table 1.

**TABLE 1:** List of materials and chemicals used in this study.

Material/Equipment	Manufacturer
×60 objective silicon oil-immersion, 1.35 N.A., 0.15 mm W.D.	Olympus, Tokyo, Japan
15 ml falcon tubes, G028-BC	Corning, Wiesbaden, Germany
50 ml falcons falcon tubes, G027-GC	Corning, Wiesbaden, Germany
6.5 mm transwell with 0.4 μm pore polyester membrane insert, 3470	Corning, Wiesbaden, Germany
Acetone	Roth, Karlsruhe, Germany
Acetylene-PEG4-Biotin, CLK-TA105-25	Jena Bioscience, Jena, Germany
AFS2	Leica, Wetzlar, Germany
Alcian Blue	Sigma-Aldrich, St. Louis, United States
Alkyne-Cy5	Jena Bioscience, Jena, Germany
Anti-fade fluorescence mounting medium – aqueous, fluoroshield	Abcam, ab104135
BSA-c	Aurion, Wageningen, Netherlands
BTTAA, CLK-067	Jena Bioscience, Jena, Germany
BX61 confocal LSM, diode laser 405 nm/647 nm	Olympus, Tokyo, Japan
Cacodylate	Serva, Heidelberg, Germany
Copper grids	Plano, Wetzlar, Germany
Coverslips 24 × 50 mm, 631-0146	VWR International, Darmstadt, Germany
CuAAC reaction ligand test kit (THPTA and BTTAA based), CLK-075	Jena Bioscience, Jena, Germany
CuSO4, CLK-MI004	Jena Bioscience, Jena, Germany
DBCO-Sulfo-Cy3, CLK-A140	Jena Bioscience, Jena, Germany
DBCO-Sulfo-Cy5, CLK-A130	Jena Bioscience, Jena, Germany
DBCO-PEG4-Biotin, CLK-A105P4	Jena Bioscience, Jena, Germany
Diamond Knife	Diatome, Nidau, Switzerland
DPBS w/o calcium and magnesium, Gibco 14190250	Thermo Fischer Scientific, Waltham, MA, United States
DPBS w/o calcium and magnesium, D8537	Sigma-Aldrich, St. Louis, United States
EM blocking solution, SKU 905.002	Aurion, Wageningen, Netherlands
Epon	Serva, Heidelberg, Germany
Falcon 24-well clear flat bottom TC-treated multiwell cell culture plate, 353047	Corning, Wiesbaden, Germany
Falcon 25 cm^2^ rectangular canted neck cell culture flask with vented cap 353109	Corning, Wiesbaden, Germany
Falcon 75 cm^2^ rectangular canted neck cell culture flask with vented cap, 353136	Corning, Wiesbaden, Germany
FluoView FV1000 V4.2 software	Olympus, Tokyo, Japan
Glutaraldehyde	Serva, Heidelberg, Germany
Goat-anti-biotin, 10 nm gold nanoparticles, SKU 110.088	Aurion, Wageningen, Netherlands
Gold nanoparticles, alkyne function, 0.05% Au 15 nm, GP15-AK-1	Nanocs, New York, United States
hAELVi cells	InSCREENeX, Braunschweig, Germany
HEPES buffer	Merck Millipore, Darmstadt, Germany
huAEC coating solution, INS-SU-1018	InSCREENeX, Braunschweig, Germany
huAEC medium, INS-ME-1013	InSCREENeX, Braunschweig, Germany
Lead citrate	Merck Millipore, Darmstadt, Germany
Lowicryl HM20	Electron Microscopy Sciences, Hatfield, United States
Methanol	Roth, Karlsruhe, Germany
Na-Ascorbate, CLK-MI005	Jena Bioscience, Jena, Germany
Nickel grids	Plano, Wetzlar, Germany
OsO4	Electron Microscopy Sciences, Hatfield, United States
Paraformaldehyde	Electron Microscopy Sciences, Hatfield, United States
Phosphotungstic acid	Merck Millipore, Darmstadt, Germany
Primocin, ant-pm-05	Invivogen, Toulouse, France
Slow scan 2K CCD camera	TRS, Moorenweis, Germany
Sucrose	Roth, Karlsruhe, Germany
THPTA, CLK-1010	Jena Bioscience, Jena, Germany
Ultramicrotome	Leica, Wetzlar, Germany
Uranyl acetate	Serva, Heidelberg, Germany
Zeiss Leo 906 electron microscope	Carl Zeiss, Oberkochen, Germany

## Methods

### Cell Culture

The immortalized alveolar epithelial cell line hAELVi was purchased from InSRCEENneX (Braunschweig, Germany). Cells were cultured on flasks or 12-well-plates that had been precoated for 2 h with a commercial coating solution harboring extracellular matrix components (Inscreenex, Braunschweig, Germany). Cells were seeded at a density of 6,000 or 8,000 cells/cm^2^ and passaged every 3 or 4 days. Medium (huAEC + Primocin 100 μg/ml) was exchanged every 2–3 days.

For experiments, cells between passage 12 and 26 have been used. Experiments were performed at an air-liquid-interface (ALI): the cell suspension (50 μl) was seeded on top of a transwell membrane system that had been precoated for 2 h, and 200–250 μl medium was added to the bottom compartment (Figures 1C,D). Medium in the bottom compartment was carefully exchanged every 2–3 days using a pipette. In order to visualize confluent cell layers, cells were seeded at a density of 6.000 cells/cm^2^ and grown for 14 days. In order to visualize cells in the exponential growth phase, cells were seeded at 8.000 cells/cm^2^ and grown for 3 days. Cells labeled with Alcian blue (see below) were grown for 3 and 10 days, respectively, and were seeded at 8.000 cells/cm^2^.

### Metabolic Glycoengineering

A summarized evaluation of different MGE concentrations and conditions applied in this study is provided in Table 2. Cells were fed with Ac_4_ManNAz or DMSO control (max. 0.1%). After incorporation of Ac_4_ManNAz, the cells were washed twice on the membrane using 50 μl DPBS, 3% BSA and subsequently, the orthogonal reaction was performed using CuAAC or SPAAC protocols.

**TABLE 2:** Summary of the different MGE experimental setups performed in this study and the corresponding outcome regarding signal-to-noise ratio.

Ac4ManNAz	Alkyne	Stabilizing agent	Signal to noise ratio	Microscopy
48 h 20 μM	Alkyne-Cy5 40 μM	THPTA	Excellent	LSM
24 h 20 μM	Alkyne-Cy5 40 μM	BTTAA	Excellent	LSM
24 h 20 μM	Alkyne-Cy5 60 μM	BTTAA	Excellent	LSM
24 h 50 μM	Alkyne-Cy5 40 μM	BTTAA	Excellent	LSM
24 h 50 μM	Alkyne-Cy5 60 μM	BTTAA	Excellent	LSM
48 h 20 μM	Alkyne-Cy5 40 μM	BTTAA	Excellent	LSM
72 h 50 μM	DBCO-Sulfo-Cy3 20 μM	N/A	Good	LSM
48 h 50 μM	DBCO-Sulfo-Cy5 60 μM	N/A	Good	LSM
48 h 50 μM	DBCO-Sulfo-Cy5 20 μM	N/A	Good	LSM
48 h 50 μM	DBCO-Sulfo-Cy5 6 μM	N/A	Mediocre	LSM
72 h 50 μM	Alkyne-gold 0.005%	BTTAA	No signal detectable	EM
72 h 50 μM	Alkyne-gold 0.001%	BTTAA	No signal detectable	EM
72 h 50 μM	Acetylene-PEG4-biotin 40 μM	BTTAA	Excellent	EM
72 h 50 μM	Acetylene-PEG4-biotin 60 μM	BTTAA	Excellent	EM
72 h 50 μM	DBCO-PEG4-biotin 60 μM	N/A	Excellent	EM

#### CuAAC

CuAAC with either BTTAA or THPTA was performed according to manufacturer’s instruction with minor modifications.

#### BTTAA

A CuSO_4_: BTTAA premix was prepared by mixing 70 μl BTTAA stock (50 mM) with 7 μl CuSO_4_ stock solution (100 mM) at RT.

The final mix (220 μl) was prepared by mixing the following reagents (all at RT) in the listed order, as this is critical for an efficient bioorthogonal reaction:

148.28 μl 100 mM Na-phosphate reaction buffer.
1.32 μl 10 mM alkyne-Cy5 or acetylene-PEG_4_-Biotin stock (final concentration 60 μM).
48.4 μl CuSO_4_: BTTAA Premix.
22 μl 1M Na-ascorbate stock solution.

#### THPTA

A CuSO_4_: THPTA premix was prepared by mixing 20 μl THPTA stock (250 mM) with 10 μl CuSO_4_ stock solution (100 mM) at RT.

The final mix (220 μl) was prepared by mixing the following reagents (all at RT) in the listed order, as this is critical for an efficient bioorthogonal reaction:

183.48 μl 100 mM Na-phosphate reaction buffer.
1.98 μl 10 mM alkyne-Cy5 stock final concentration (60 μM).
13.2 μl CuSO_4_: THPTA Premix.
22 μl 1M Na-ascorbate stock solution.

Whenever lower concentrations of the alkyne were used, a reciprocal amount of Na-phosphate buffer was added to achieve a volume of 220 μl.

For (ultimately futile) attempts to label with alkyne-gold, the following two final mixes using alkyne-gold in a 1:19 dilution (for BTTAA) or 1:9 dilution (for THPTA) were prepared:

75.6 μl 100 mM Na-phosphate reaction buffer.
6 μl alkyne-gold.
26.4 μl CuSO_4_: BTTAA Premix.
12 μl 1M Na-ascorbate stock solution.

or

133.4 μl 100 mM Na-phosphate reaction buffer.
23 μl alkyne-gold.
50.6 μl CuSO_4_: THPTA Premix.
23 μl 1M Na-ascorbate stock solution.

In addition, a mix using alkyne-gold in a 1:49 dilution was tested—to exclude the possibility that the biorthogonal reactions failed due to inappropriate buffer concentrations. However, also using that dilution we could not detect any gold nanoparticles (data not shown).

The final alkyne mix (50 μl per transwell) was pipetted on top of the cells and incubated at 37°C for 30 min.

#### SPAAC

SPAAC was performed using either DBCO-Sulfo-Cy5, DBCO-Sulfo-Cy3 or DBCO-PEG_4_-Biotin:

DBCO-“X” was diluted in DPBS, 1% FBS to the required final concentration. The solution (50 μl per transwells) was added on top of the cells and incubated at 37°C for 1 h.

### Fluorescence Microscopy Sample Preparation

Cells were washed once using 50 μl DPBS, 3% BSA before fixation. Cells were fixed with DPBS containing 4% paraformaldehyde for 15 min at 37°C. To this end, the fixative was added to the top (100 μl) and bottom compartment (500 μl). Subsequently, cells were washed three times using DPBS on a shaker. Cells were counterstained using 0.1% DAPI in DPBS for ∼10 min on a shaker in the dark. After washing twice with DPBS, the transwell membrane harboring the cells was cut out with fine scissors and mounted on a glass slide using liquid mounting medium (Figure 1E). Two small magnets were applied to the glass slide and the coverslip, opposing each other, in order to gently flatten the membrane (Figures 1E,F). This step is critical for visualizing the cell layer as otherwise the transwell membrane tends to get embedded tangentially and appears wavy. The embedded transwells were visualized by confocal laser scanning microscopy using a BX61 (Olympus) and a × 60 objective, silicon oil-immersion, 1.35 N.A., 0.15 mm W.D. Both, Cy5 and DAPI were excited using a multi-argon laser (405 nm/633 nm). Cy3 was excited using a helium-neon laser (559 nm). Images were acquired with FluoView FV1000 V4.2 software using the preconfigured settings for DAPI, Cy3 and Cy5, respectively.

### Transmission Electron Microscopy (TEM) Sample Preparation

For standard cytochemical fixation/Epon embedding, cells were washed twice using 50 μl DPBS, 3% BSA and once using 50μl DPBS. After washing, cells were fixed by adding 0.15 M HEPES buffer containing 1.5% paraformaldehyde and 1.5% glutaraldehyde for at least 3 h (up to 24 h) at 4°C. To this end, fixative was added to the top (100 μl) and bottom compartment (500 μl). Post fixation was performed using 1% OsO_4_ in 0.1 M cacodylate buffer at RT for 2 h, followed by incubation in half-saturated aqueous uranyl acetate over night at 4°C. After dehydration in a graded acetone series, samples were transferred to Epon resin. Ultrathin sections (70 nm) were prepared using an ultramicrotome equipped with a diamond knife, collected on pioloform-coated copper grids and subsequently stained with lead citrate according to Reynolds (Reynolds, 1963).

In order to stain cell-surface glycans, Alcian blue was optionally added to the fixative (0.15%) and to every subsequently applied solution (0.075%) until 70% acetone was reached within the graded series.

For freeze substitution and immunogold labeling, cells were fixed using a mixture of 4% paraformaldehyde and 0.1% glutaraldehyde in 0.2 M HEPES buffer. After cryoprotective infiltration using 2.3 M sucrose in PBS, cells were frozen in liquid nitrogen and transferred to a freeze substitution system (AFS2, Leica, Wetzlar, Germany). Freeze substitution was performed at −90°C using methanol containing 0.5% uranyl acetate at −90°C. Subsequently, samples were embedded in Lowicryl HM20 at −45°C (Fehrenbach and Ochs, 1998). Ultrathin sections (70 nm) were collected on pioloform-coated nickel grids and used for immunogold labeling.

Sections were floated on drops of 50 mM glycine, 0.1% BSA-c in PBS (PH 7.4) for 20 min and subsequently blocked for 30 min using blocking solution (Aurion, Wageningen, Netherlands). After a washing step in incubation buffer (0.1% BSA-c in PBS), sections were labeled for 90 min at RT using an antibody against Biotin, conjugated with 10 nm gold particles, diluted 1:30 in incubation buffer Finally, the sections were rinsed in incubation buffer (six times) and PBS (three times), fixed with 2.5% glutaraldehyde in PBS and treated with an aqueous solution of 4% uranyl acetate and 1% phosphotungstic acid (5 min). Finally, grids were washed three times in distilled water.

Grids were examined on a Zeiss Leo 906 electron microscope at 80 kV acceleration voltage, and electron micrographs were recorded using a slow scan 2K CCD camera (TRS, Moorenweis, Germany).

## Results

### Visualization of Sialic Acid by Fluorescence Microscopy

**FIGURE 2:**
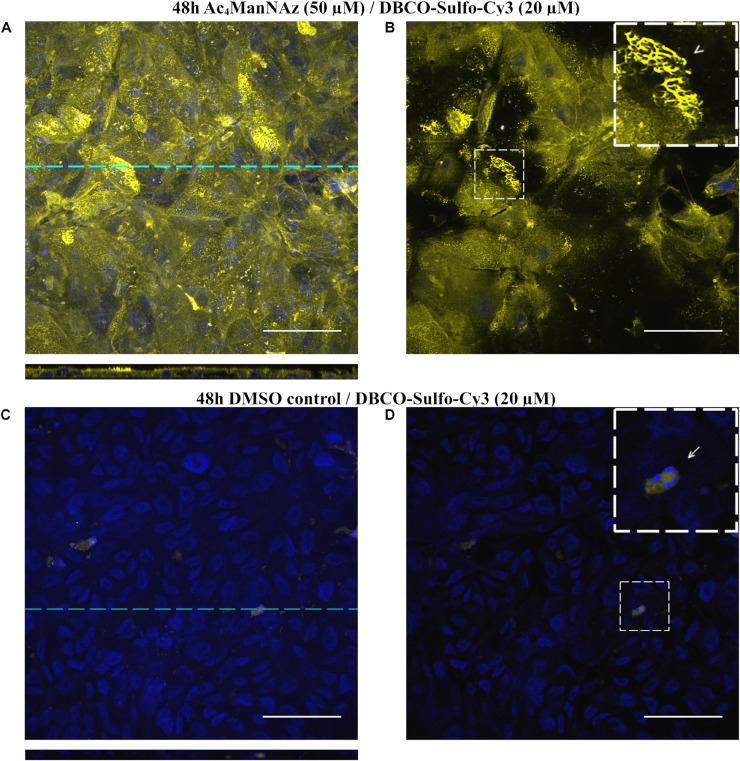
LSM micrographs of hAELVi cells that have been grown for 14 days after seeding. Cells have been fed for 48 h with Ac_4_ManNAz (20 μM) or DMSO control and stained with alkyne-Cy5 (40 μM) using Cu(I) stabilizing helper THPTA. **(A)** Full z-stack projection of hAELVI cells. Dashed line indicates position of the corresponding cross-section plane. **(B)** Single section plane of **(A)** at the apical side of the cells shows membranous staining. Inset shows apical part of two cells at higher magnification. Arrowhead points to longitudinally oriented microvilli, while the fine dots covering most of the apical cell bodies (asterisk) represent transversally oriented microvilli. **(C)** Full z-stack projection of hAELVi cells fed with DMSO control display no alkyne-Cy5 staining indicating specificity of the staining. **(D)** Single section of **(C)** at a very basal position. The inset displays a higher magnification. The arrow points to the 0.4 μm pores of the transwell membrane which stain, because DAPI accumulates in the pores and cannot be washed out efficiently. Scale bars: 50 μm. Cross sections are scaled in *z*-axis by factor 3 to provide better visibility.

For visualization of sialic acid as part of the alveolar epithelial glycocalyx, we used the recently established human alveolar epithelial cell line hAELVi, derived from immortalized primary alveolar epithelial cells. Mimicking the physiological situation in the alveolar space, we grew hAELVi cells at an air-liquid-interface (ALI) using polyethylene transwell membranes (∅0.4 μm pore size) that had been coated with extracellular matrix components. After 2 weeks, hAELVi cells form a dense layer that display intact barrier function (Kuehn et al., 2016). We fed hAELVi cells with Ac_4_ManAz (20 μM) for 48 h, an unnatural precursor metabolite of the sialic acid biosynthesis pathway. Metabolically labeled sialic acids were finally visualized by chemical linkage to alkyne-Cy5 (40 μM, 30 min) in a Co(I) catalyzed azide-alkyne click chemistry reaction (CuAAC) along with THPTA as a helper substance. Subsequently, cells were fixed, counterstained with DAPI and the membrane was cut and mounted for laser scanning microscopy (LSM). Fourteen days old cells presented with intense plasma membrane staining which allowed the visualization of microvilli (Figures 2A,B). The staining was particularly pronounced at the apical (luminal) cell surface (Figure 2A, see cross-section projection). As expected, cells that had been fed with DMSO control did not display any detectable Cy5 staining (Figure 2C), highlighting the specificity of CuAAC. Of note, DAPI gathers within the 0.4 μM pores of the polyethylene membrane leading to artificial staining of the basal pores, which are, however, clearly distinguishable in size from the cell nuclei (Figure 2D). Another potential pitfall concerns the embedding of the membrane that tends to corrugate within the mounting medium impairing planar scanning. We overcame this issue by sandwiching the mounted membrane between two small magnets.

Next, we tested different concentrations of Ac_4_ManAz (20–50 μM) and alkyne-Cy5 (40–60 μM) in 3 days old hAELVi cells using BTTAA as a helper substance of CuAAC (Supplementary Figure S1). We confirmed excellent signal-to-noise ratio even at low concentrations, respectively. When visualized 3 days after plating, hAELVi cells were less confluent, larger in shape and formed long filopodia (thin protrusions) that extended up to ∼100 μm, often spanning from cell to cell. Although present, microvilli were less frequently seen.

In addition to visualizing the glycocalyx using CuAAC, we also performed copper-free strain-promoted azide-alkyne cycloaddition (SPAAC) using DBCO-Sulfo-Cy3 (Supplementary Figure S2) or DBCO-Sulfo-Cy5 (not shown). DBCO-based cycloaddition overcomes the potential cytotoxicity of copper making it suitable for long term *in vitro* and *in vivo* live-cell imaging at the expense of higher background staining due to non-specific binding to thiol groups. We found best and very acceptable signal-to-noise ratio when cells were fed with at least 50 μM Ac_4_ManAz and stained with 20 μM DBCO-Sulfo-fluorophore. Considerable background was detected within single apoptotic cells as defined by highly condensed chromatin and fragmented nuclei (Supplementary Figure S2D). Of interest, in addition to regular cell membrane staining, we occasionally detected intensely stained structures of tubular appearance forming an extracellular meshwork (Supplementary Figure S2B).

### Visualization of Sialic Acid Could Not Be Achieved Using Alkyne-Gold Nanoparticles

In order to investigate the distribution of sialic acid in more detail, we decided to combine metabolic glycoengineering with ultrastructural analyses providing resolution at the single molecule level. To this end, we fed hAELVi cells with Ac_4_ManNAz, but replaced alkyne-Cy5 by a solution of alkyne-gold nanoparticles for detectability by EM. Subsequently, cells were fixed, embedded and contrasted using an EPON-based electron microscopy protocol that had been optimized for ultrastructural preservation of lung tissue.

To our disappointment, we could not detect any gold nanoparticles although cells treated in parallel for confocal microscopy stained effectively (Supplementary Figure S3). We consider that the inevitable dilution (1:9) of the alkyne-gold solution during reagent preparation—which leads to a final concentration of 0.005% (w/v) of gold nanoparticles corresponding to calculated 0.93 nM alkyne-gold—is probably too low for efficient detection. This may become particular limiting in EM, as ultrathin sections contain far less labeled molecules per section when compared to an optical section plane in confocal microscopy.

### Alcian Blue Enhances a Standard EM Protocol for Visualization of the Glycocalyx

**FIGURE 3:**
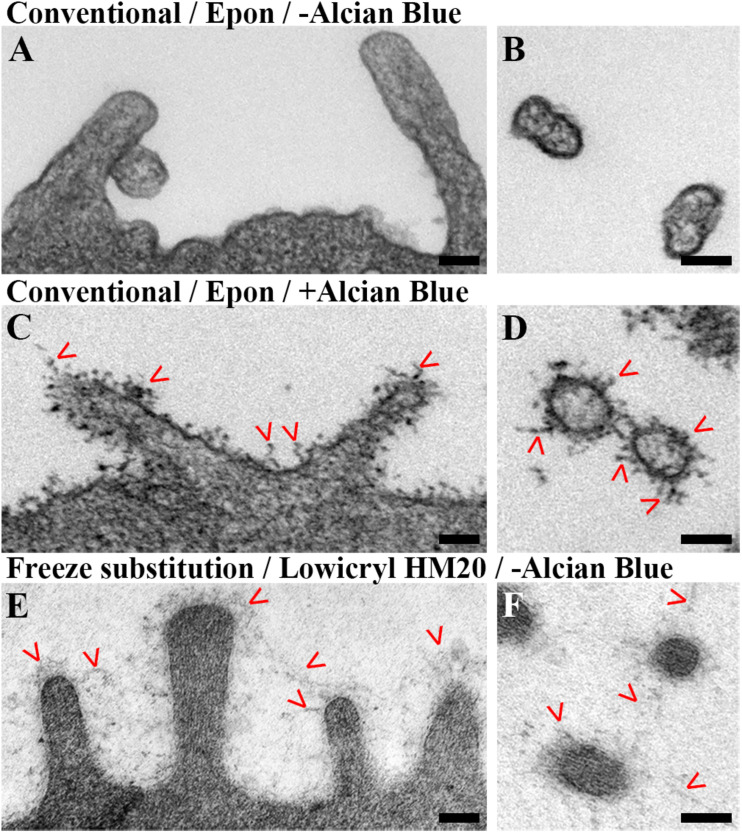
Visualization of glycocalyx in electron micrographs of hAELVi cells using different EM procedures. **(A,B)** Chemically fixed and EPON embedded hAELVi cells 3 days after seeding. **(A)** Longitudinally, **(B)** transversally sectioned microvilli poorly displayed any ultrastructurally recognizable glycocalyx labeling. **(C,D)** Addition of Alcian blue labels glycocalyx as branching structures in **(C)** longitudinally and **(D)** transversally sectioned microvilli. **(E,F)** Mild chemical fixation and freeze substitution of 14 days old hAELVi cells resolves a fine, interconnected network structure around **(E)** longitudinally and **(F)** transversally sectioned microvilli. Scale bars: 100 nm.

Despite the expected good ultrastructural preservation of intracellular structures, we noticed that the Epon embedding protocol performed poorly in visualizing the glycocalyx in general (Figures 3A,B). In order to evaluate whether the basic protocol either leads to the complete decomposition of the glycocalyx or simply fails in contrasting it sufficiently, we added Alcian blue during fixation and sample processing as it has previously been used in enhancing labeling of the glycocalyx by non-specific binding to glycosaminoglycans (Ruggeri et al., 1975; Singh et al., 2007). Indeed, adding Alcian blue to our Epon embedding protocol contrasted the glycocalyx as visualized by the typical ramifying structures (up to ∼150 nm in length) emerging on top of the plasma membrane, especially in microvilli-rich domains (Figures 3C,D). However, when we compared our EPON-embedding to a freeze-substitution protocol using Lowycryl HM20 (HM20), we found the latter to preserve the network structure of the glycocalyx in more detail, even in the absence of additional labeling agents (Figures 3E,F).

### Visualization of Sialic Acid by Combining MGE With a Biotin/AB-Gold Labeling Approach in a Freeze-Substitution Protocol

**FIGURE 4:**
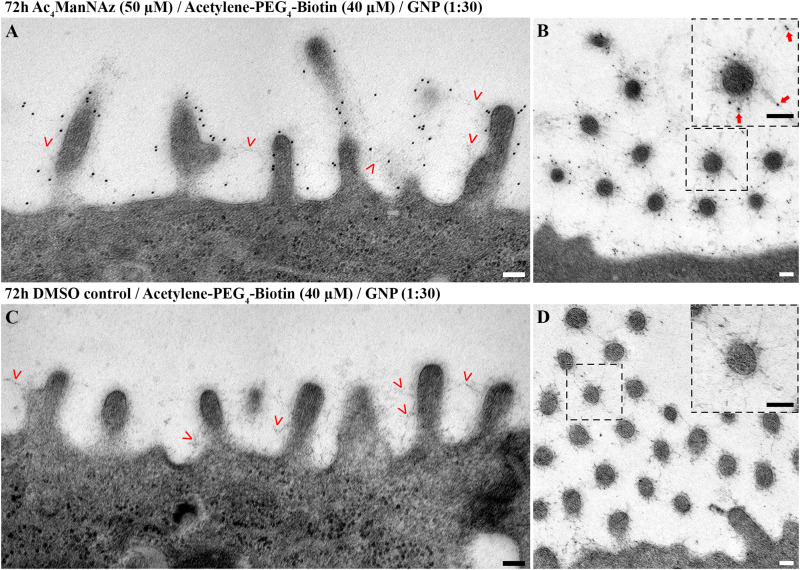
Gold nanoparticles label sialic acid and colocalize with glycocalyx on the plasma membrane and around microvilli. Electron micrographs of hAELVi cell samples prepared using mild chemical fixation and subsequent FS. Micrographs show 14 days old cells fed **(A,B)** with Ac_4_ManNAz (50 μM) or **(C,D)** DMSO control for 72 h before reacting with Acetylene-PEG_4_-Biotin (40 μM). Postembedding, antibodies against biotin (dilution 1:30) linked to 10 nm gold nanoparticles were applied. Microvilli sectioned **(A)** longitudinally and **(B)** transversally display extensive labeling with gold nanoparticles within the glycocalyx. Those often colocalize at the distal end of branched filamentous structures **(B)**. Cells fed with DMSO control display no attached gold nanoparticles at the membrane, at **(C)** longitudinally and **(D)** transversally sectioned microvilli despite presence of glycocalyx networks, indicating specificity of the labeling. Scale bars: 100 nm. Insets: Large insets derive from micrographs recorded at higher magnification. Arrowheads: arrowheads point to fuzzy material on top of the plasma membrane that represents glycocalyx.

As HM20 based freeze-substitution technically allows for post-embedding antibody staining, we decided to modify the alkyne click chemistry reaction in favor of a combined click-chemistry antibody-based approach. For this purpose, we labeled cells with Acetylene-PEG_4_-Biotin (CuAAC), before fixation and subsequent freeze substitution using HM20. After sectioning, we applied an antibody against biotin linked to a gold nanoparticle (anti-Biotin-AB-Au). This strategy successfully detected labeled sialic acid molecules at an ultrastructural level with high specificity (Figure 4 and Supplementary Figures S4, S5). We confirmed the results in an SPAAC setting using DBCO-PEG_4_-Biotin and the same antibody (Supplementary Figure S6).

Anti-Biotin-AB-Au specifically colocalized with the network structures of the glycocalyx, i.e., within close proximity to the plasma membrane (up to ∼200 nm). Similar to our findings from confocal microscopy, labeled sialic acid as well as glycocalyx was most prominent around microvilli. Around sectioned microvilli (Figure 4B and Supplementary Figure S5A) and along the membrane (Supplementary Figure S5), we could detect sialic acid frequently localized at the distal end of glycan structures, corresponding to a terminal position.

**FIGURE 5:**
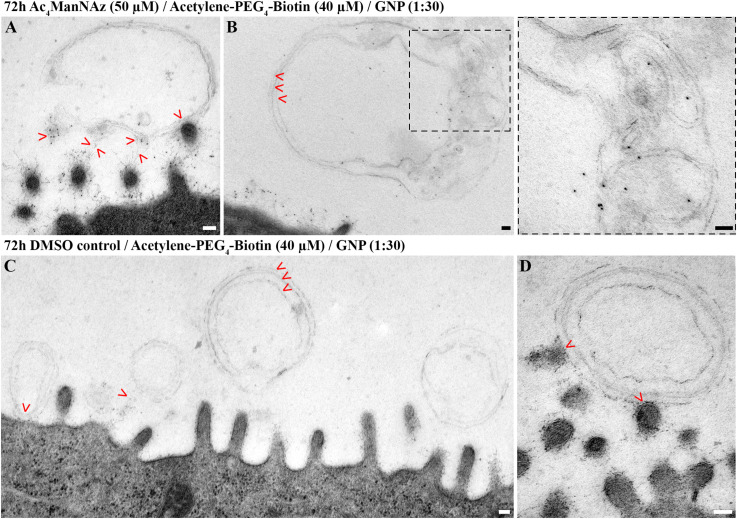
hAELVi cells secrete lamellar membranous structures that interact with microvilli and were labeled for sialic acid. Electron micrographs of hAELVi cells prepared using a mild chemical fixation and subsequent FS. Micrographs show 14 days old cells that had been fed **(A,B)** with Ac_4_ManNAz (50 μM) or **(C,D)** DMSO control for 72 h before reacting with Acetylene-PEG_4_-Biotin (40 μM). Postembedding, antibodies against biotin (dilution 1:30) linked to 10 nm gold nanoparticles were applied. **(A)** Microvilli sectioned transversally interacting with multilamellar membranous structure. Gold nanoparticle labeling is displayed at the zone of contact (arrowheads). **(B)** Multilamellar membranous material forming higher ordered structures in close proximity to the plasma membrane. Arrowheads point to three lamellae, respectively. Inset: Large inset derives from micrograph recorded at higher magnification and shows gold nanoparticles within the multilamellar structure, which are devoid of obvious glycocalyx-like fibrous material. **(C)** In cells treated with DMSO control, attached gold particles are neither visible around the microvilli nor at or within the secreted lamellar membranous structures (arrowheads) indicating specificity of the labeling. **(D)** Micrograph recorded at higher magnification. Arrowheads point to the contact zone between a multilamellar membranous structure and multiple microvilli, apical of cells treated with DMSO control. Scale bars: 100 nm.

In close proximity to the microvilli, we could further detect unilamellar and multilamellar membranous structures (Figure 5). Those often appeared to be attached to the fine glycan network of the glycocalyx (Figures 5A,C,D). Upon labeling of sialic acid, such areas were positive for gold nanoparticles (Figure 5A). Furthermore, we detected gold nanoparticles deeply within the lamellar structures, and this was notably in the absence of structurally visible colocalizing glycocalyx networks (Figure 5B). Some of the multilamellar structures adopted signs of a higher degree of organization (Figure 5B, Inset).

Cells fed with DMSO control displayed glycocalyx as well as uni- and multilamellar structures, yet in neither of those we could detect attaching gold nanoparticles, demonstrating the specificity of anti-Biotin-AB-Au labeling (Figures 5C,D).

## Discussion

In the last decades, glycan biology has made considerable progress in systematically applying established techniques in combination with newly emerging tools (Varki, 2017). For surface labeling of distinct species of sialic acids, sugarbinding lectins have been proven successful in light- and electron microscopy (Taatjes et al., 1988; Sata et al., 1991; Langereis et al., 2015). However, lectins rely on multivalency of their ligands as their single-site affinity is typically poor (Kiessling and Grim, 2013; Taylor et al., 2015). Thus, lectin binding tends to reflect the presence of closely clustered residues, inflicting bias in terms of sensitivity and quantification. In contrast to lectin based approaches, labeling of sialic acids originating from Ac_4_ManNAz (Figure 1A) first, allows for single molecular analysis independent of ligand multivalency and second, enables the study of all types of sialic acids present on the surface, independent of the sub-entity of sialic acids for which lectins are specific. Interestingly, acetylated 4-Azido-ManNAc as an alternative to Ac_4_ManNAz has been shown to integrate predominantly into sialic acids of O-glycans (Moller et al., 2012). Thus, MGE has also potential to label distinct sialic acid subspecies with certain specificity. Regarding single-molecule detection methods of glycans, there is a current lack of studies using *in vitro* or even *in vivo* settings (Lakshminarayanan et al., 2018). Super-resolution microscopy has been applied to study surface glycans (Letschert et al., 2014; Chen et al., 2016), and achieves a resolution of ∼20 nm, which is at the edge of being suitable for visualizing protein complexes (ribosome: >80 proteins = 25 nm). This resolution is, however, still not sufficient for analysis of single glycan residues.

The herein established combination of MGE and EM proved suitable to overcome several of the before mentioned limitations, thus allowing for the first time for visualization of specific alveolar epithelial glycocalyx components at the single molecule level. Yet, care should be taken when utilizing MGE labeling in quantitative approaches, as the rate of metabolic incorporation of the labeled precursors has to be taken into consideration.

So far, glycocalyx-like structures of hAELVi cells have been previously demonstrated in one published electron micrograph of a hAELVi/THP-1 co-culture system (Kletting et al., 2018). In this study, the surface coat was non-specifically visualized around microvilli of hAELVi cells that seemingly interacted with THP-1 cells by a protocol using chemical fixation followed by Epon embedding and a combination of osmium and ferrocyanide treatment. Ferrocyanide, which was not used in the present study, had previously been reported to be able to trace the glycocalyx of liver and renal cortex when applied in appropriate concentrations (Neiss, 1984). In our hands, hAELVi cells showed a staining pattern comparable to Kletting et al. (2018) when adding Alcian blue to our Epon embedding protocol (Figure 3), thus independently validating the presence of cell surface glycans. Alcian blue has positive charges, and is therefore able to bind to various acidic sugars when applied at low osmolarity conditions, most importantly sulfated and sialyated residues but also non-sulfated hyaluronic acid (Danes et al., 1970; Mohos and Skoza, 1970; Hayat, 1993). Upon binding, it typically forms particles (2–3 nm) with variable contrast, that can arrange in rods or chains, which showed a subtle degree of branching in our study. As such, we could improve a regular chemical fixation/Epon embedding method that was originally optimized for ultrastructural preservation of lung alveoli but not suitable to visualize the glycocalyx of hAELVi cells, with the purpose of labeling glycan structures non-specifically.

As described in the introduction, there is ongoing debate in the field, how the type of fixation, subsequent dehydration steps and chemical labeling reagents affects the preservation of the glycocalyx. Recently, it was hypothesized that dehydration rather than fixing is critically in terms of volume collapse of the glycocalyx as chemical fixation/dehydration versus cryofixation/freeze-substitution (which includes dehydration) resulted in comparable heights within lung- and heart vessels that were perfused with lanthanum and dysprosium (LaDy) for chemical labeling of the glycocalyx (Hempel et al., 2020). In this study, the authors then tried visualizing the glycocalyx of vessels by combining cryofixation and low-dose x-ray tomography (SXT), a methodology requiring a synchrotron but no dehydration step. However, this method provided not enough contrast for the glycocalyx to be detected without additional chemical labeling. Adding the latter (LaDy) prior to cryofixation/SXT partly overcame this issue but was not suitable to resolve any structural details due to its very high absorbance of x-rays. Despite, the authors indirectly estimated the height of the glycocalyx by measuring the distance between the endothelial plasma membrane and trapped erythrocytes and found larger dimensions as compared to dehydration depending methods.

Here, we present the first ultrastructural analysis of the hAELVi cell glycocalyx based on a mild chemical fixation with subsequent freeze substitution procedure (Figure 4 and Supplementary Figures S4, S5). Our freeze substitution protocol is devoid of additional glycan labeling chemicals (like LaDy or Alcian blue) and allowed for a more unbiased representation of the glycocalyx ultrastructure. According to previous findings (Reipert et al., 2018), our results show freeze substituted cells presenting with a fine, fuzzy surface coat (height up to ∼200 nm), slightly exceeding dimensions found for Alcian blue labeling (up to ∼150 nm). Albeit fainter in contrast, the network structure generally displayed a higher degree of complexity and interconnectivity.

We detected antibody-linked gold nanoparticles specifically bound to metabolically labeled sialic acid throughout the glycocalyx of the plasma membrane, using CuAAC (Figure 4 and Supplementary Figures S4, S5) and SPAAC (Supplementary Figure S6), respectively. Importantly, we could localize gold particles at the end of branched filaments (Figure 4BSupplementary Figure S5), which correlates with the biochemically defined terminal position of sialic acid at the end of glycoconjugates (Varki et al., 2015). This prompts us to argue, that the preservation of the glycocalyx using our freeze substitution procedure holds promise to reflect the ultrastructural architecture to a reasonable extent. Our findings thus indicate that the combination of MGE with our freeze substitution protocol preserved a plausible representation of the glycocalyx that is unbiased of additional glycan stabilizing chemicals while equally providing molecular identity to the labeled structures in alveolar epithelial cells for the first time.

The recently established hAELVi cell line has been described as a model for ATI cells, although molecular characterization has been first, limited to the expression of a few marker genes and second, not been performed using cells harvested at ALI (Kuehn et al., 2016). After 13–14 days of culture at ALI, we report extensive formation of microvilli and a well-marked glycocalyx, both structural features more prevalent in ATII cells (Brooks, 1969; Adamson and Bowden, 1970; Roth, 1973; Mason, 2006; Ochs et al., 2020). Thus, the differentiation fate of the cell line might depend on the applied culture conditions. Of note, a comprehensive ultrastructural and molecular characterization is beyond the scope of the present study which focuses on establishing methods of glycocalyx visualization in alveolar epithelial cells. At the moment, there is ongoing refinement of hAELVi cell culture medium aiming to improve the physiological properties of the model (Tobias May, InSRCEENneX GmbH, personal communication, 28th September 2020).

Our ultrastructural analysis revealed hAELVi cells to present uni- and more complex multilamellar membranous structures (Figure 5). We regularly observed these structures to be in direct contact with the glycocalyx on top of the plasma membrane. Previous work studying interactions between surfactant and specific glycocalyx components has been limited to the proteoglycan hyaluronan (Lu et al., 2005; Taeusch et al., 2005; Wang et al., 2006; Lopez-Rodriguez et al., 2013; Dong et al., 2020). By combining MGE and EM, we found the extracellular lamellar structures to be enriched in sialic acids (Figure 5). We thus can hypothesize, that either glycolipids or glycoproteins are present in these lamellar structures. Notably, glycoproteins in alveolar spaces such as surfactant protein A (SP-A) (Munakata et al., 1982; White et al., 1985; Haagsman et al., 1991; Berg et al., 2000)contain sialic acid. Recently, glycans on surfactant protein D (SP-D), isolated from human amniotic fluid, were similarly reported to be sialylated (Arroyo et al., 2020). Future work will focus on characterizing the membranous material secreted by hAELVi cells in more detail, potentially opening novel model qualities for this cell line.

In summary, we established hAELVi cells as a model to study the alveolar epithelial cell glycocalyx and report a combination of tools for its ultrastructural characterization with molecular identity. As MGE is applicable for *in vivo* studies, this approach will facilitate the identification of the evolutionary developed functionalities of the so far scarcely investigated alveolar epithelial glycocalyx, its role in the interaction with surfactant components in the lining layer, and alterations upon onset of diseases.

## Data Availability Statement

The original contributions presented in the study are included in the article/Supplementary Material, further inquiries can be directed to the corresponding author/s.

## Author Contributions

EL-R, RB, ST, and MO evolved study design and conception and contributed to data interpretation. RB and ST performed the experiments. RB, JG, and ST wrote the manuscript. CH contributed to providing material. WK and JK offered constructive suggestions for the research. EL-R, CH, WK, and JK contributed to script revision. All authors approved to the final version.

## Conflict of Interest

The authors declare that the research was conducted in the absence of any commercial or financial relationships that could be construed as a potential conflict of interest.

## Supplementary Material

Supplementary Figure 1 LSM micrographs of hAELVi cells 3 days after seeding. Cells have been fed for 24 h with different concentrations of Ac_4_ManNAz (20 μM, 60 μM, or DMSO control) and stained with alkyne-Cy5 using Cu(I) stabilizing helper BTTAA. **(A)** 20 μM Ac_4_ManNAz, 40 μM alkyne-Cy5, laser intensity (LI) 2%, **(B)** 20 μM Ac_4_ManNAz, 60 μM alkyne-Cy5, LI 2%, **(C)** 50 μM Ac4ManNAz, 40 μM alkyne-Cy5, LI 1%, **(D)** 50 μM Ac_4_ManNAz, 60 μM alkyne-Cy5, LI 1%, **(E)** 60 μM alkyne-Cy5 LI 1%, **(F)** 60 μM alkyne-Cy5 LI 2%. Yellow channel: alkyne-Cy5, blue channel: DAPI. Laser intensities indicated for yellow channels. Scale bars: 50 μm.

Supplementary Figure 2 LSM micrographs of hAELVi cells that have been grown for 14 days after seeding. Cells have been fed for 72 h with Ac_4_ManNAz (50 μM) and stained with DBCO-Sulfo-Cy3 (20 μM) in a SPAAC. **(A)** Full z-stack projection of hAELVI cells. Dashed line indicates position of the corresponding cross-section plane. **(B)** Single section plane of **(A)** at an apical position of the cells. Inset displays a higher magnification. The arrowhead points to an intensely stained structure (compare also to cross section plane of **A**) at the apical side of a cell that forms a tubular meshwork. **(C)** Full z-stack projection of hAELVI cells fed with DMSO control display minor background staining. **(D)** Single section plane of **(C)**. Inset displays a higher magnification. The arrow points to a dying cell with fragmented nuclei and highly condensed chromatin which displays considerable amount of unspecific DBCO-Sulfo-Cy5 staining. Scale bars: 50 μm. Cross sections are scaled in z-axis by factor 3 to provide better visibility.

Supplementary Figure 3 hAELVi cells that have been grown for 14 days after seeding. Cells, seeded the same day, had been fed with Ac_4_ManNAz (20 μM) for 48 h **(A–C)** or 50 μM for 72 h **(D)** and labeled with alkyne-Cy5 (40 μM) **(A–C)** or alkyne-gold (0.005%) **(D)** using CuAAC stabilizing helper substance BTTAA. **(A)** Full z-stack projection of hAELVi cells. Dashed line indicates position of the corresponding cross-section plane. **(B)** Single section plane of **(A)** at the apical side of the cells shows membranous staining. Inset shows apical part of cells at higher magnification. Brackets points to longitudinally oriented microvilli, while the fine dots covering most of the apical cell bodies represent transversally oriented microvilli. **(C)** Full z-stack projection of hAELVi cells fed with DMSO control display no alkyne-Cy5 staining indicating specificity of the staining using CuAAC. **(D)** Electron micrograph of chemically fixed and EPON embedded cells showing longitudinally sectioned microvilli that display no gold nanoparticles labeling around the microvilli or other parts of the plasma membrane indicating failing labeling efficiency using alkyne-gold. Scale bars LSM: 50 μm. Cross sections are scaled in z-axis by factor 3 to provide better visibility. Scale bars EM: 100 nm.

Supplementary Figure 4 Sialic acid labeling of the glycocalyx on plasma membrane and around microvilli using CuAAC and gold nanoparticles. Electron micrograph (freeze substitution protocol) of 14 days old hAELVi cells fed **(A,B)** with Ac_4_ManNAz (50 μM) or **(C,D)** DMSO control for 72 h before reacting with Acetylene-PEG_4_-Biotin (60 μM). Postembedding, antibodies against biotin (dilution 1:30) linked to 10 nm gold nanoparticles were applied. Microvilli sectioned **(A)** longitudinally and **(B)** transversally show extensive labeling by gold nanoparticles, colocalizing with extracellular fuzzy material that exhibit network-like structures. Cells fed with DMSO control display no attached gold nanoparticles at the membrane, at **(C)** longitudinally or **(D)** transversally sectioned microvilli despite presence of glycocalyx networks, indicating specificity of the labeling. Scale bars: 100 nm.

Supplementary Figure 5 Sialic acid labeling of the glycocalyx around microvilli **(A)** and on plasma membrane **(B,C)** using CuAAC and gold nanoparticles. Electron micrograph (freeze substitution protocol) of 14 days old hAELVi cells fed with Ac_4_ManNAz (50 μM) for 72 h before reacting with Acetylene-PEG_4_-Biotin (40 μM). Postembedding, antibodies against biotin (dilution 1:30) linked to 10 nm gold nanoparticles were applied. Note that a large proportion of gold nanoparticles appears to be localized at the distal end of glycan structures. Scale bars: 100 nm.

Supplementary Figure 6 Labeling of sialic acid around the plasma membrane of microvilli using SPAAC and gold nanoparticles. Electron microscopy using freeze substitution protocol of 14 days old hAELVi cells fed **(A)** with Ac_4_ManNAz (50 μM) or **(B)** DMSO control for 72 h before reacting with DBCO-PEG_4_-Biotin (60 μM). Postembedding, antibodies against biotin (dilution 1:30) linked to 10 nm gold nanoparticles were applied. Scale bars: 100 nm.

## Abbreviations

ATI: alveolar epithelial type I cell
ATII: alveolar epithelial type II cell
BTTAA: 2-(4-((Bis((1-(*tert*-butyl)-1*H*-1,2,3-triazol-4-yl)methyl)amino)methyl)-1*H*-1,2,3-triazol-1-yl)acetic acid
CuAAC: Cu(I) catalyzed azide-alkyne cycloaddition
DBCO: dibenzocyclooctyne
EM: electron microscopy
GNP: gold nanoparticles
LaDy: mixture of lanthanum and dysprosium
LSM: laser scanning microscopy
MGE: metabolic glycoengineering
PEG: polyethylene glycol
RT: room temperature
SPAAC: strain-promoted-azide-alkyne cycloaddition
THPTA: Tris((1-hydroxy-propyl-1H-1,2,3-triazol-4-yl)methyl)amine.
