# Supplementary figures and images for: Metabolic Glycoengineering Enables the Ultrastructural Visualization of Sialic Acids in the Glycocalyx of the Alveolar Epithelial Cell Line hAELVi
Source: Front Bioeng Biotechnol. 2021 Jan 14;8:614357. doi: 10.3389/fbioe.2020.614357 (PMC7841390; doi:10.3389/fbioe.2020.614357)

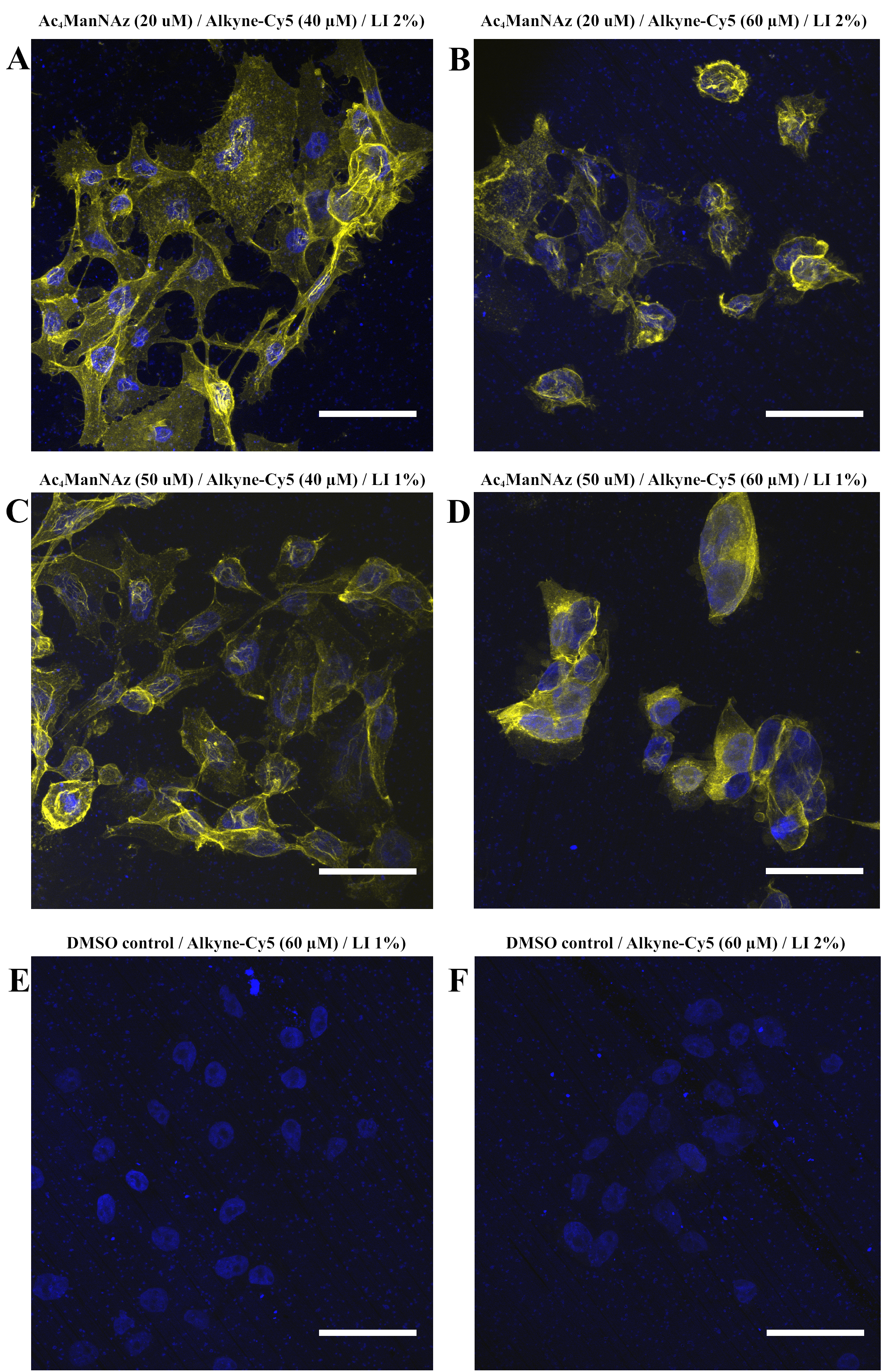

Supplement: Supplementary Figure 1 — LSM micrographs of hAELVi cells 3 days after seeding. Cells have been fed for 24 h with different concentrations of Ac4ManNAz (20 μM, 60 μM, or DMSO control) and stained with alkyne-Cy5 using Cu(I) stabilizing helper BTTAA. (A) 20 μM Ac4ManNAz, 40 μM alkyne-Cy5, laser intensity (LI) 2%, (B) 20 μM Ac4ManNAz, 60 μM alkyne-Cy5, LI 2%, (C) 50 μM Ac4ManNAz, 40 μM alkyne-Cy5, LI 1%, (D) 50 μM Ac4ManNAz, 60 μM alkyne-Cy5, LI 1%, (E) 60 μM alkyne-Cy5 LI 1%, (F) 60 μM alkyne-Cy5 LI 2%. Yellow channel: alkyne-Cy5, blue channel: DAPI. Laser intensities indicated for yellow channels. Scale bars: 50 μm. [file Image_1.TIFF]

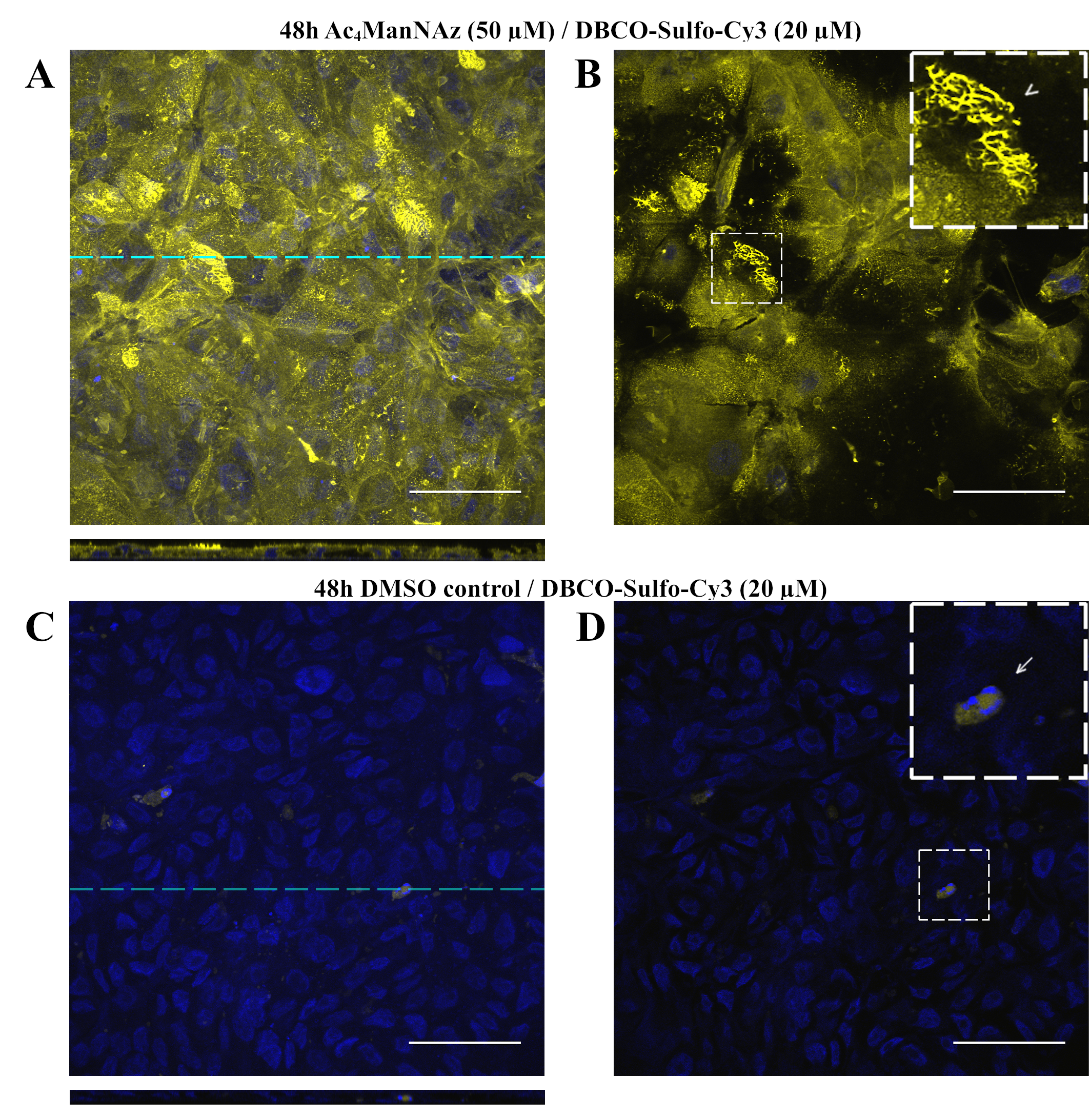

Supplement: Supplementary Figure 2 — LSM micrographs of hAELVi cells that have been grown for 14 days after seeding. Cells have been fed for 72 h with Ac4ManNAz (50 μM) and stained with DBCO-Sulfo-Cy3 (20 μM) in a SPAAC. (A) Full z-stack projection of hAELVI cells. Dashed line indicates position of the corresponding cross-section plane. (B) Single section plane of (A) at an apical position of the cells. Inset displays a higher magnification. The arrowhead points to an intensely stained structure (compare also to cross section plane of A) at the apical side of a cell that forms a tubular meshwork. (C) Full z-stack projection of hAELVI cells fed with DMSO control display minor background staining. (D) Single section plane of (C). Inset displays a higher magnification. The arrow points to a dying cell with fragmented nuclei and highly condensed chromatin which displays considerable amount of unspecific DBCO-Sulfo-Cy5 staining. Scale bars: 50 μm. Cross sections are scaled in z-axis by factor 3 to provide better visibility. [file Image_2.tiff]

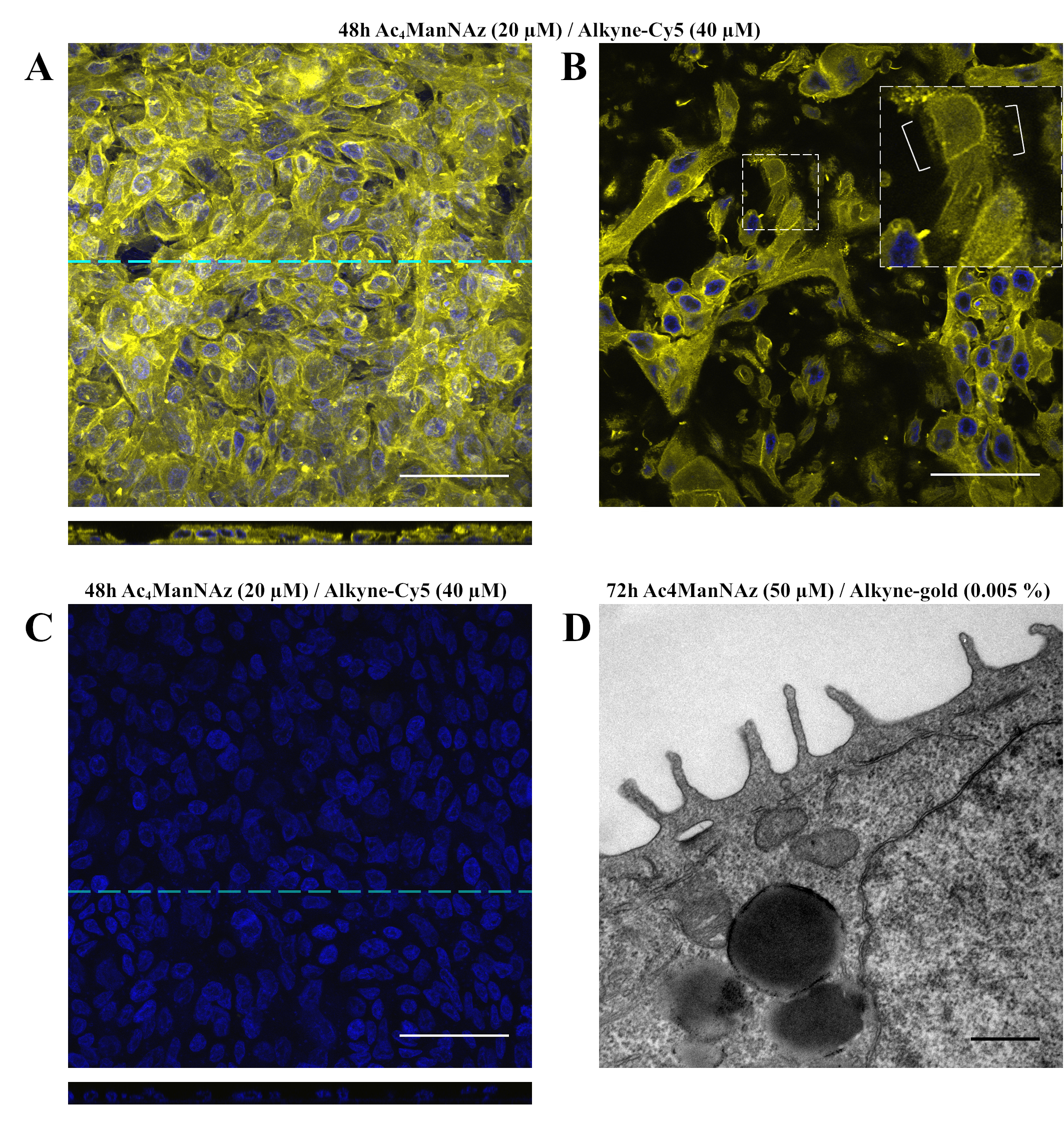

Supplement: Supplementary Figure 3 — hAELVi cells that have been grown for 14 days after seeding. Cells, seeded the same day, had been fed with Ac4ManNAz (20 μM) for 48 h (A–C) or 50 μM for 72 h (D) and labeled with alkyne-Cy5 (40 μM) (A–C) or alkyne-gold (0.005%) (D) using CuAAC stabilizing helper substance BTTAA. (A) Full z-stack projection of hAELVi cells. Dashed line indicates position of the corresponding cross-section plane. (B) Single section plane of (A) at the apical side of the cells shows membranous staining. Inset shows apical part of cells at higher magnification. Brackets points to longitudinally oriented microvilli, while the fine dots covering most of the apical cell bodies represent transversally oriented microvilli. (C) Full z-stack projection of hAELVi cells fed with DMSO control display no alkyne-Cy5 staining indicating specificity of the staining using CuAAC. (D) Electron micrograph of chemically fixed and EPON embedded cells showing longitudinally sectioned microvilli that display no gold nanoparticles labeling around the microvilli or other parts of the plasma membrane indicating failing labeling efficiency using alkyne-gold. Scale bars LSM: 50 μm. Cross sections are scaled in z-axis by factor 3 to provide better visibility. Scale bars EM: 100 nm. [file Image_3.TIFF]

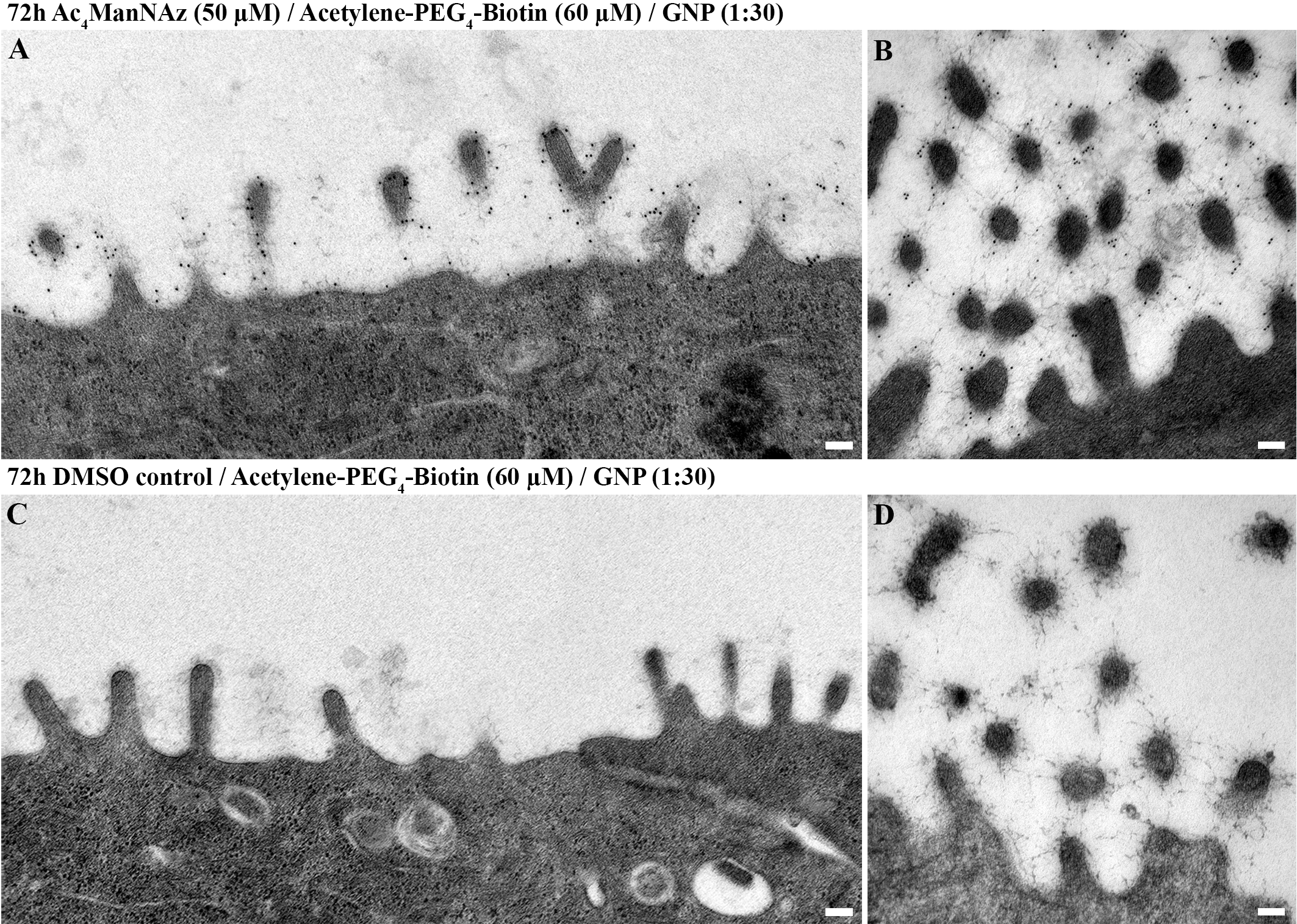

Supplement: Supplementary Figure 4 — Sialic acid labeling of the glycocalyx on plasma membrane and around microvilli using CuAAC and gold nanoparticles. Electron micrograph (freeze substitution protocol) of 14 days old hAELVi cells fed (A,B) with Ac4ManNAz (50 μM) or (C,D) DMSO control for 72 h before reacting with Acetylene-PEG4-Biotin (60 μM). Postembedding, antibodies against biotin (dilution 1:30) linked to 10 nm gold nanoparticles were applied. Microvilli sectioned (A) longitudinally and (B) transversally show extensive labeling by gold nanoparticles, colocalizing with extracellular fuzzy material that exhibit network-like structures. Cells fed with DMSO control display no attached gold nanoparticles at the membrane, at (C) longitudinally or (D) transversally sectioned microvilli despite presence of glycocalyx networks, indicating specificity of the labeling. Scale bars: 100 nm. [file Image_4.TIF]

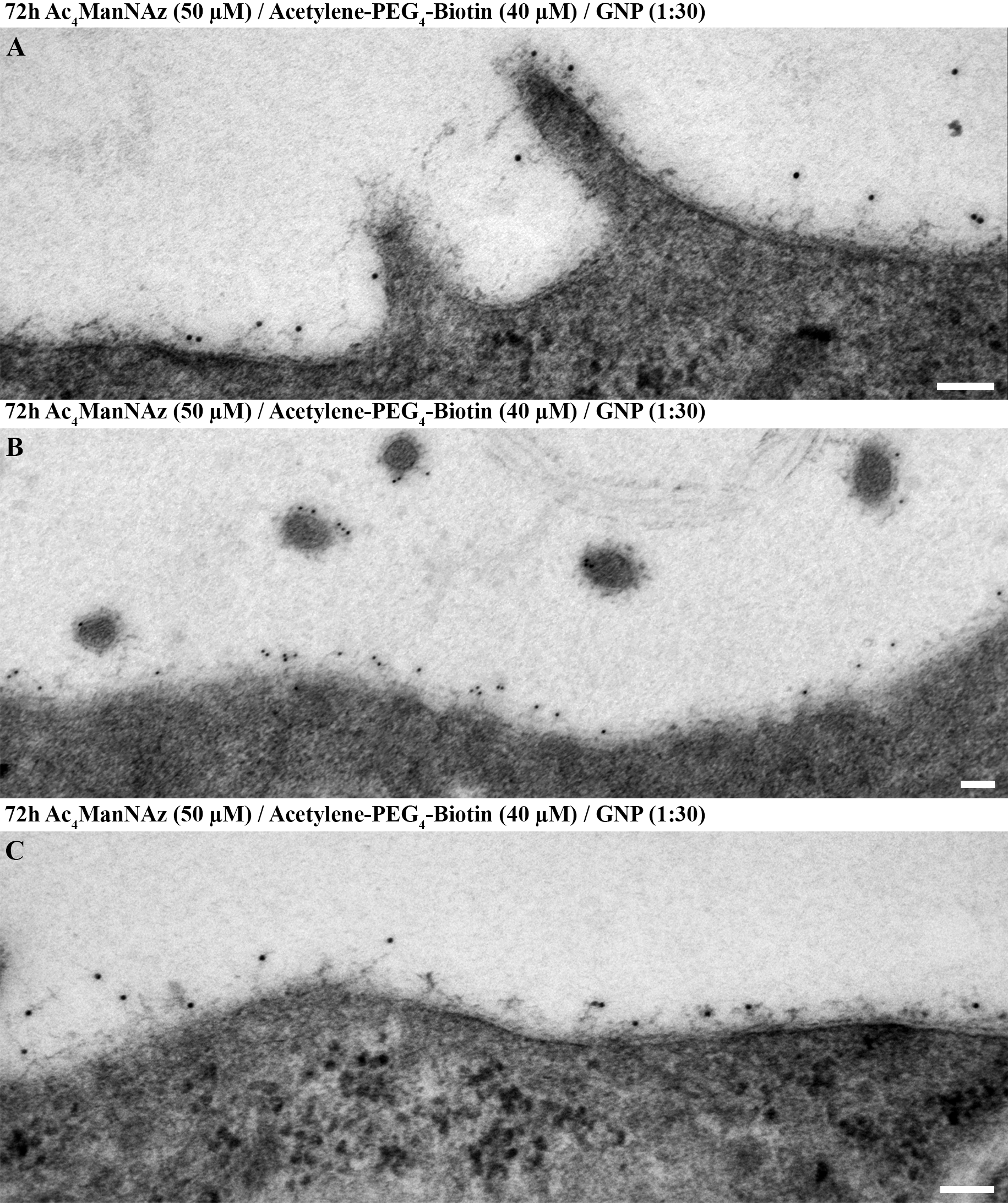

Supplement: Supplementary Figure 5 — Sialic acid labeling of the glycocalyx around microvilli (A) and on plasma membrane (B,C) using CuAAC and gold nanoparticles. Electron micrograph (freeze substitution protocol) of 14 days old hAELVi cells fed with Ac4ManNAz (50 μM) for 72 h before reacting with Acetylene-PEG4-Biotin (40 μM). Postembedding, antibodies against biotin (dilution 1:30) linked to 10 nm gold nanoparticles were applied. Note that a large proportion of gold nanoparticles appears to be localized at the distal end of glycan structures. Scale bars: 100 nm. [file Image_5.TIF]

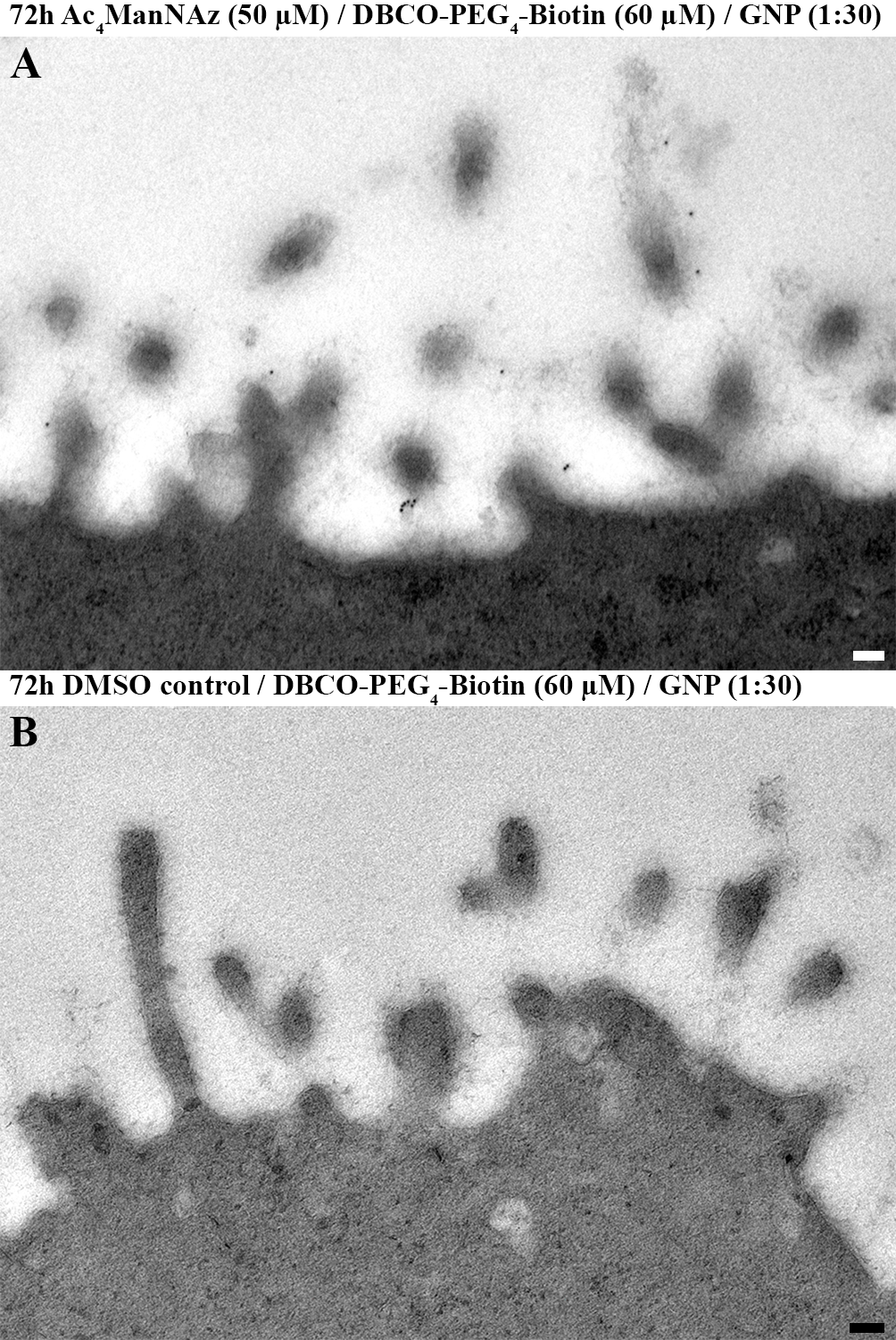

Supplement: Supplementary Figure 6 — Labeling of sialic acid around the plasma membrane of microvilli using SPAAC and gold nanoparticles. Electron microscopy using freeze substitution protocol of 14 days old hAELVi cells fed (A) with Ac4ManNAz (50 μM) or (B) DMSO control for 72 h before reacting with DBCO-PEG4-Biotin (60 μM). Postembedding, antibodies against biotin (dilution 1:30) linked to 10 nm gold nanoparticles were applied. Scale bars: 100 nm. [file Image_6.TIF]
